# Relationship between nursing home COVID-19 outbreaks and staff neighborhood characteristics

**DOI:** 10.1371/journal.pone.0267377

**Published:** 2022-04-19

**Authors:** Karen Shen

**Affiliations:** Department of Economics, Harvard University, Cambridge, MA, United States of America; Cardiff University, UNITED KINGDOM

## Abstract

The COVID-19 pandemic has been particularly deadly for residents of nursing homes and other long-term care facilities. This paper analyzes COVID-19 deaths at nursing homes during the first wave of the pandemic in the United States during the spring and early summer 2020. By combining data on facility-level COVID-19 deaths during this period with data on the neighborhoods where nursing home staff reside for a sample of eighteen states, this paper finds that staff neighborhood characteristics were a large and significant predictor of COVID-19 nursing home deaths. Even after controlling for the county where a facility is located, one standard deviation increases in average staff neighborhood (Census tract) population density, public transportation use, and non-white share were associated with 1.3 (p < .001), 1.4 (p < .001), and 0.9 (p < .001) additional deaths per 100 beds, respectively. These effects are larger than all facility management or quality variables, and larger than the effect of the nursing home’s own neighborhood characteristics. These results suggest COVID-19 outbreaks in staff communities can have large consequences for the facilities where they work, even in highly-rated facilities, and that disparities in nursing home outbreaks may be related to differences in the types of neighborhoods nursing home staff live in.

## Introduction

The COVID-19 pandemic’s overwhelming impact on nursing homes in the United States and worldwide has been well-documented [1]. As of November 22, 2020, of the 256,597 deaths from COVID-19 nationwide, the Center for Medicare and Medicaid Services (CMS) had recorded 72,642 were among nursing home residents. This number would already imply that 28% of deaths have been among nursing home residents; however, due to the fact that federal reporting was optional before May 10 and the possibility that facilities may have under-reported deaths even after May 10, it is likely to be a significant underestimate [2].

Numerous hypotheses have emerged about what factors affected the vulnerability of facilities to infection, with some pointing to poor management or infection control procedures, and others to specific actions and policies such as the timing of when nursing homes became locked down to visitors, state policies governing the transfer of recovering COVID-positive patients to nursing homes, and the supply of personal protective equipment (PPE) and testing [3,4]. Another possibility is that outbreaks are unpredictable and unpreventable and that luck and geography, rather than factors under a nursing home operator or policymaker’s control, largely determined which facilities saw outbreaks and which did not.

In support of the latter hypothesis, several studies have documented the importance of county infection rates in predicting COVID-19 cases and deaths at nursing homes [5,6], while most studies have not found a relationship of outbreaks with star rating or even infection control violations [5,7,8]. However, there has been considerably less evidence on whether and how much geography may matter at finer granularities, particularly as compared to other facility variables such as ownership or star rating. This type of evidence is important because it may be better able to highlight or explain disparities that exist across facilities in the same area who were thus subject to the same macro-level policies and conditions (e.g. stay-at-home orders, mask mandates, weather, and distance from initial hot spots).

The objective of this study was to examine the relationship between nursing home COVID-19 outbreaks and differences in the characteristics of the residential neighborhoods of each nursing home staff, controlling for the nursing home’s county, using an eighteen-state sample and a novel measure of nursing home staff neighborhoods. These results have the potential to support and extend other literature that has documented the importance of staff transmission in COVID-19 outbreaks [9], as well as to shed light on potential mechanisms to explain disparities in the impact of COVID-19 on different facilities that have been documented in the media and in other studies [6,10].

## Methods

### Data

The universe of study is all Medicare and Medicaid-certified skilled nursing facility (hereafter, “nursing home”) in the eighteen sample states, defined using the CMS Nursing Home Compare database. This database provides the name and address of every certified nursing home, as well as a unique provider identifier number that allows it to be matched to other data sources.

The main outcome of interest is facility-level COVID-19 deaths during the first wave of the pandemic (before July 10, 2020). This deaths data is compiled from data published by each state’s department of health between July 5 and July 10, 2020. The eighteen states in the sample were selected primarily due to availability of this data. State data is needed because the federal data did not require facility reporting before May 17, 2020, and is therefore missing a significant number of deaths. S1 File in the supplementary material discusses this data in detail, including differences across the sample states in their reporting requirements. Because these data contained facility names, but not facility identifiers, S1 File also describes the fuzzy-matching and geocoding techniques I used to match facilities in the state data to their federal provider identifier. S1 Fig shows the timing of the data relative to the trends in total COVID-19 deaths and COVID-19 deaths among nursing home residents, as well as the timing of the federal data. This figure shows that the data occur around a local minimum in the total deaths trend—thus why we refer to this as the “first wave”—and also reveals the need for state data to study this wave, as the federal data appears to begin after most deaths in the wave had already occurred.

The Longitudinal Employer-Household Dynamics Origin-Destination Employment Statistics (LODES) data from the U.S. Census Bureau was used to measure which neighborhoods each facility’s staff live in. These data are primarily derived from state administrative records (e.g. unemployment insurance records), and commonly used to study commuting patterns. To the best of my knowledge, this is the first paper to leverage these data to measure the residential neighborhoods of nursing home staff.

The LODES Origin-Destination file provides counts of employment for every work and home census block pair in three large industry groupings (“goods producing”, “trade/transportation/utilities”, “all other services”). Staff neighborhood measures were constructed by averaging the residence census tract characteristics of all workers employed on the nursing home’s census block in the “all other services’” category (although the LODES data provides the home census block for each worker, the neighborhood characteristics of interest are only available at the tract level). The validity of these measures are based on the assumption that the neighborhoods of service employees on a nursing home’s census block will be representative of the neighborhoods of the nursing home’s employees. For many blocks, I hypothesize that this is likely to be true by default because a nursing home may be the only or largest source of service employment on a block. Two checks of the data support this hypothesis. First, using the Workplace Area Characteristics file from the LODES, which provides employment counts for each workplace census block in twenty (rather than three) industry groupings, I estimate that on the median block in my sample, 92% (IQR: [62%, 100%]) of service employment is in the healthcare and social assistance sector. Second, to consider the possibility that there may be other healthcare or social assistance employers that are not the nursing home on the same block (one example of this is nursing homes that are collocated with hospitals), I use an estimate of the ratio of nursing home employment to nursing home residents derived from national estimates (1.23 employees to every 1 resident) to calculate a predicted employment count for each facility. I find that on the median block, this predicted number is 58% (IQR: [37%, 91%]) of the service sector employment on the block.

For blocks where the nursing home does not comprise the majority of service-sector employment, the results will be affected only if the other service sector employees live in different types of neighborhoods than the nursing home employees. There may be reasons that this is true—for example, nursing home workers may have different demographic characteristics than other service sector employees—and reasons that it is unlikely to be true, such as if neighborhood employment patterns are heavily influenced by public transportation routes. Since it is not possible to assess this directly, in the results section, I test the robustness of my findings to excluding facilities whose predicted employment is significantly below the actual service sector employment on the block. A final potential source of error is due to the timing of the LODES data, which is published with a significant lag. The most recent data at the time of writing is a snapshot of all workers on April 1, 2017 and is thus unlikely to capture the exact neighborhoods of nursing home workers because of worker turnover [11] or moves, but may be representative of the type of neighborhoods in which the nursing home’s workers are likely to reside if, for example, nursing home hiring practices, commuting routes, or peer effects create persistent variation in staff neighborhood characteristics.

I match each of the service sector residence census blocks to tract-level estimates (the finest geography available) from the American Community Survey (ACS) 5-year estimates (2014–2018) of population density, poverty, income, and use of public transportation, and employment by industry and occupation. The employment data is used to compute a predicted share of “frontline workers,” following other literature [12,13]. Finally, weighted averages of these characteristics are taken at the facility-level for all tracts where staff live to obtain the final measures of facility staff exposures.

I collect several additional facility-level variables that may be important in explaining COVID-19 deaths to include in my analysis. Data on the most recent star ratings, ownership (for-profit, non-profit, or public), occupancy rate, and prior infection control-related violations are obtained from the Nursing Home Compare database. I also collect data on chain ownership from the Online Survey Certification and Reporting and Certification and Survey Provider Enhanced Reporting (OSCAR/CASPER) data (facility data collected by state survey agencies during annual LTCF certification inspections), resident demographics including the share of residents whose primary source of payment is Medicaid and the share of residents who are non-white from the Brown LTC Focus Database—which aggregates data from the Minimum Data Set—and data on average wages paid to Certified Nursing Assistants (CNAs) and Registered Nurses (RNs) from the Healthcare Cost Report Information System (HCRIS) data from CMS.

Data on county case counts are obtained from the New York Times COVID-19 Database, which compiles data from state and local governments and health departments to produce daily cumulative case counts at the county level since the beginning of the pandemic. In addition, to help investigate the mechanisms through which local staff residence geography may affect facility deaths, I also collect data on population case rates at finer geographies (town, zip or tract) was also collected from a subsample of eight state health departments that released this data.

### Statistical analyses

The main analyses are ordinary least squares regressions where the dependent variable is the cumulative number of reported COVID-19 deaths at a facility as of early July per 100 beds. Deaths are used because they are likely to be less dependent on testing and more consistently measured across facilities. Independent continuous variables are scaled to have a standard deviation of 1 to allow for comparisons of effect sizes, while binary variables are unchanged. All regressions contain county fixed effects, which serve to control for county-specific policies and local COVID-19 exposure. The estimates on neighborhood effects should thus be interpreted as within-county effects; however, the interpretation of these effects may vary depending on county size. In general, the independent variables chosen are standard to the literature, and cross-correlations of all independent variables of interest were computed in order to avoid issues of multi-collinearity. The tract characteristics shown represented our pre-analysis hypotheses, so we do not adjust for multiple comparisons. Regression analyses were conducted using Stata 16.1. The facility-level data on deaths and tract-level neighborhood characteristics used in this paper are available at DOI 10.5281/zenodo.4308760.

## Results

**Fig 1:**
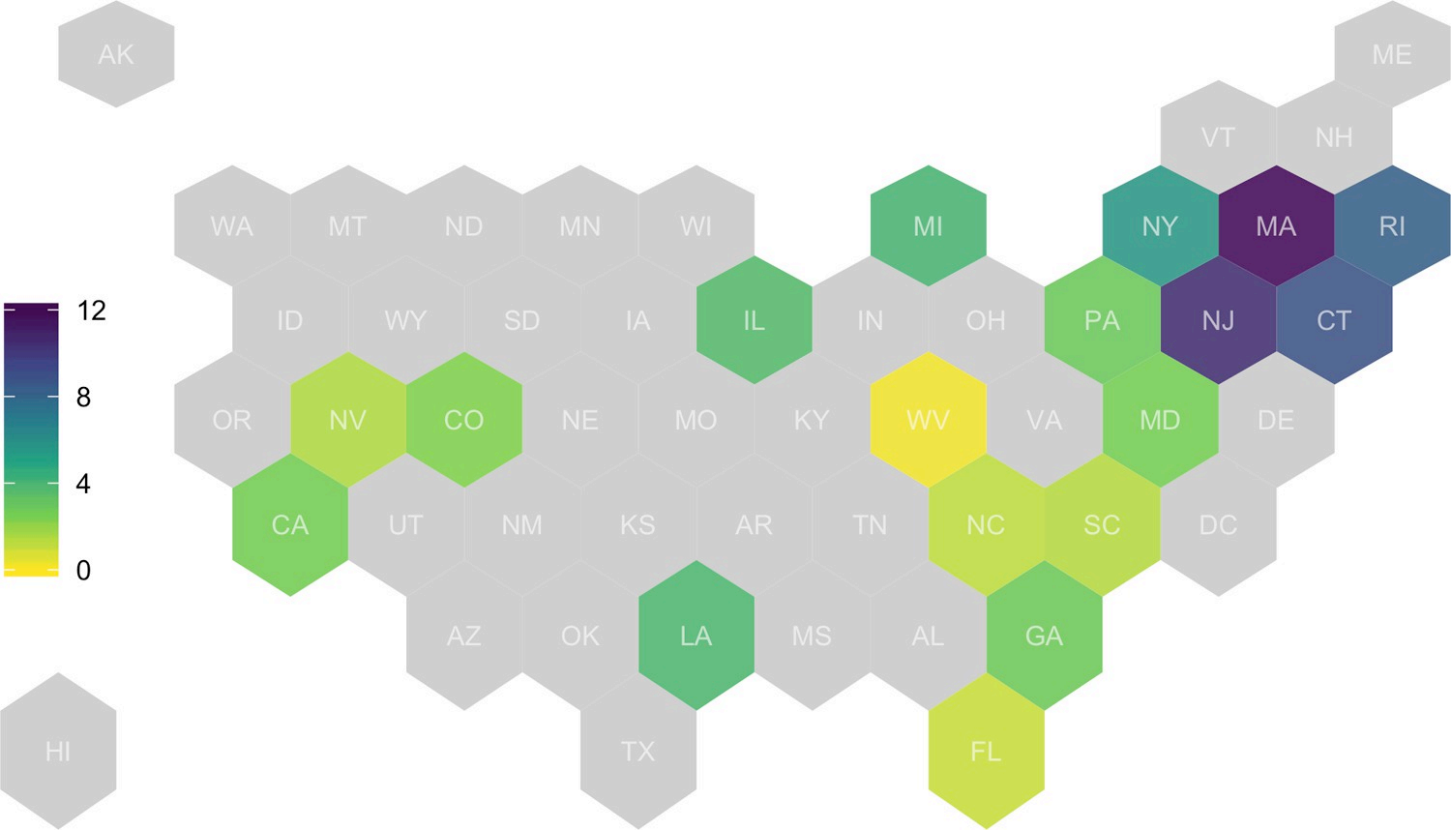
Average of facility-level first-wave COVID-19 deaths per bed in sample states, by state. States are shaded according to the average number of deaths per bed at nursing homes in each state. States not included sample are in light grey. N = 6,132.

**Fig 2:**
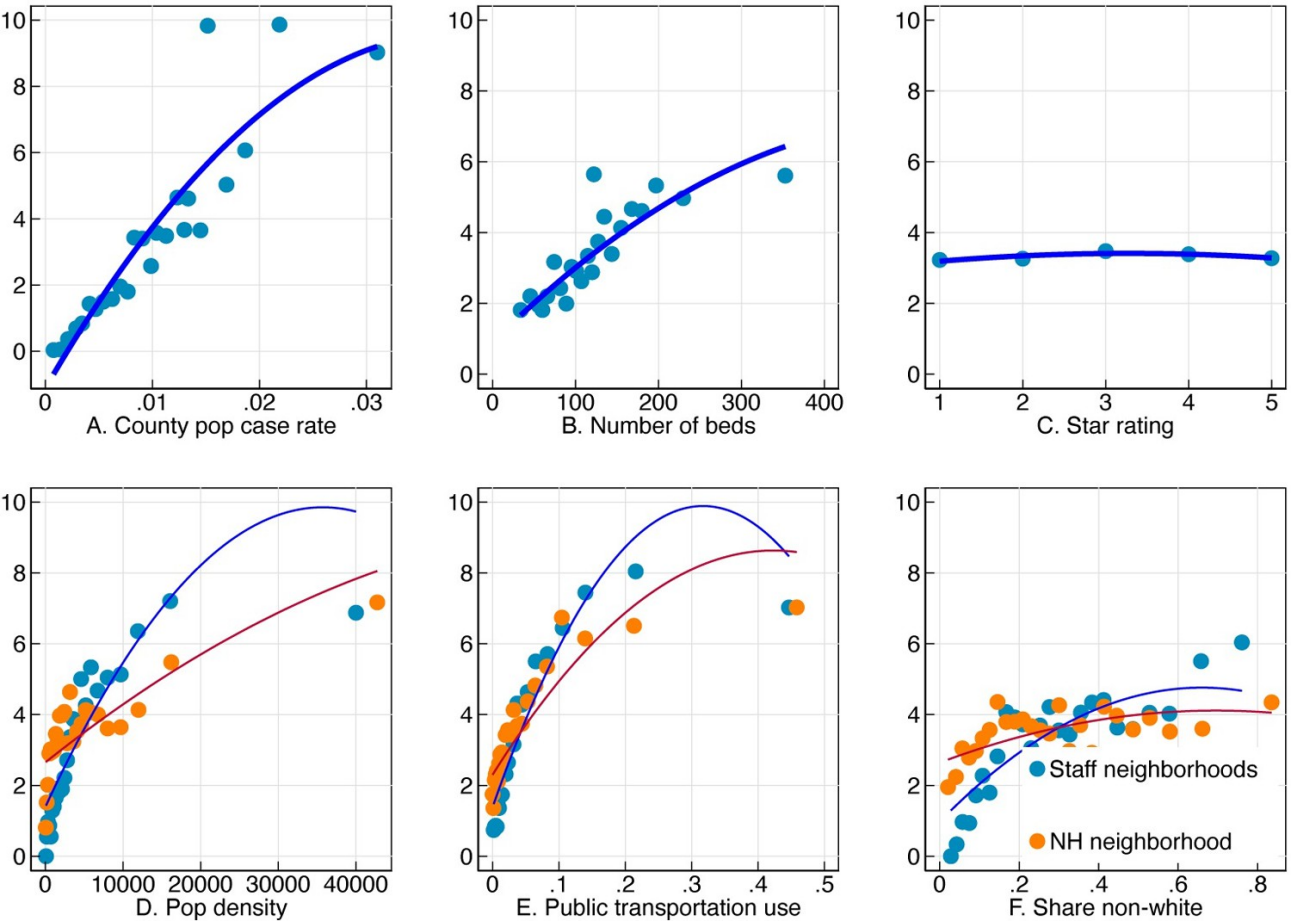
Relationship of facility deaths per bed with staff and nursing home neighborhood characteristics, facility size, and star rating. Each panel of the figure shows average nursing home deaths per 100 beds on the y-axis binned by a different variable along the x-axis. Bins are of equal sizes, and the line represents a quadratic fit.

Fig 1 introduces the eighteen state sample used in this paper. The sample includes states from each region of the country. The impact of the first wave of the pandemic on these states varied substantially. Fig 2 summarizes the main outcome variable (facility deaths per 100 beds) by state. Overall, at the time of the data, the average nursing home in this sample had experienced 3.7 deaths per bed. For some states in the Northeast (MA, NJ, CT, RI), this number was more than 8 deaths per bed, while some states in other regions (WV, FL, NC, SC, NV) had experienced fewer than 2 deaths per bed. This variation is roughly in line with variation in total deaths during the first wave of the pandemic, but some of these differences may be because states varied in their reporting requirements. Since the main analysis controls for each facility’s county, these differences in reporting should have less impact on the main results.

**Fig 3:**
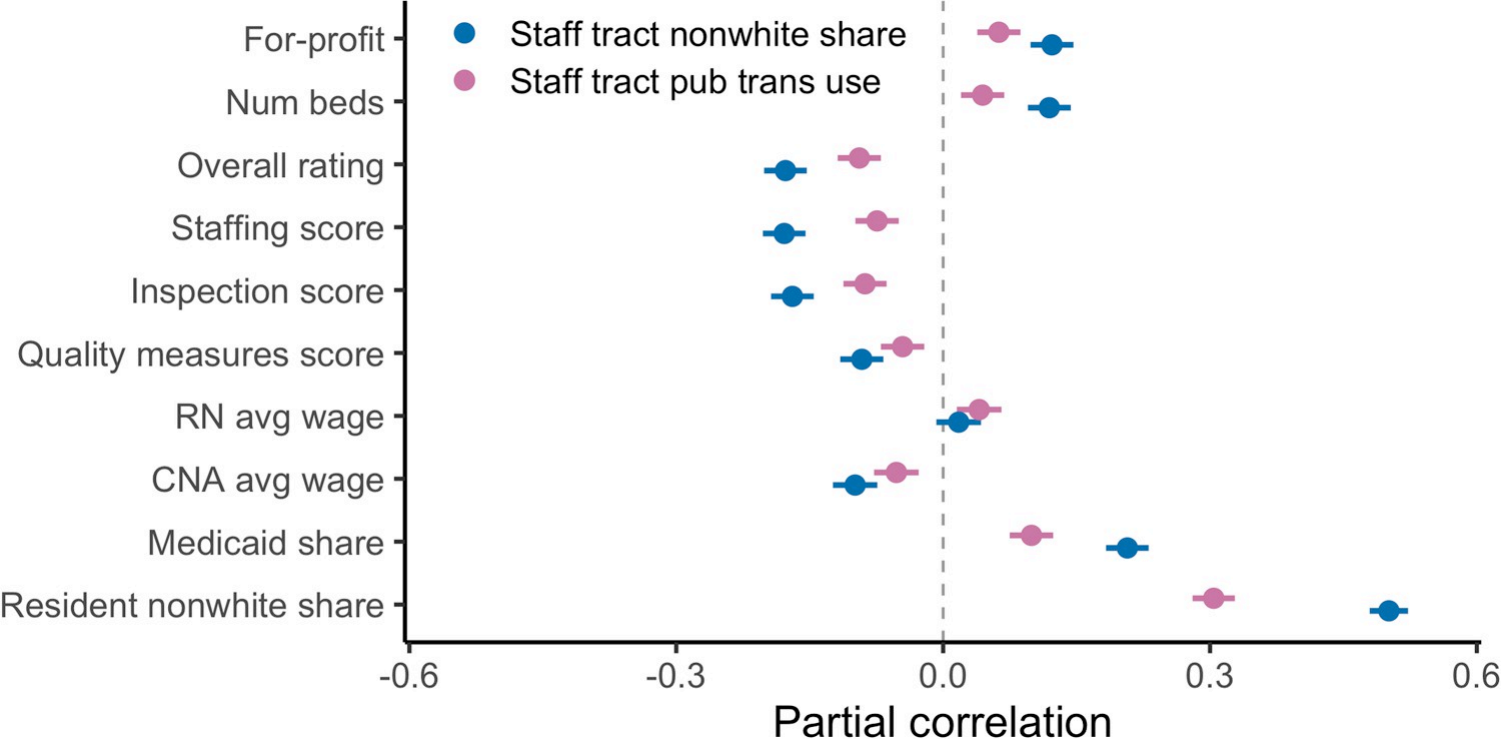
Partial correlations between staff neighborhood characteristics and other facility characteristics, controlling for county fixed effects. This figure reports partial correlations of different facility characteristics listed along the y-axis with two neighborhood characteristics of interest: Public transportation use and non-white share. Correlations control for county fixed effects. Lines represent 95% confidence intervals.

Fig 2 shows how the number of deaths per bed at a facility is related to a set of variables of interest. Nursing home outbreak sizes are strongly related to the county infection rate (Panel A), as well as the number of beds in the facility (Panel B). Panel C plots average death rates by star rating and finds virtually no relationship between the two variables. The bottom row of Fig 3 shows the relationship of facility deaths to staff neighborhood characteristics. Nursing homes are grouped by the population density, public transportation use (share of workers who commute to work on public transportation), and non-white share of the census tract where their staff live (in blue) and the tract where they are located (in orange). Facility outbreaks are strongly associated with all three of these characteristics, with steeper slopes for staff neighborhoods than the nursing home neighborhood in each case.

Table 1 presents models that jointly estimate the effect of facility characteristics and staff and nursing home neighborhood characteristics on facility deaths per bed. As of early July, there had been an average of 3.7 deaths per 100 beds across all facilities in the sample summary statistics for the independent variables are provided in S1 Table). The model in column (1) does not include any neighborhood characteristics, and shows that for-profit nursing homes are associated with an additional .53 deaths (SE .52, p = .007) and nursing homes that belong to chains are associated with an additional .35 deaths (SE .16, p = .033) per 100 beds. As seen above, even after scaling deaths by the number of beds in a facility, facility size continues to be an important factor in explaining death rates, as are occupancy rates, which may reflect both the mechanical effect of more residents per bed in the facility and potential crowding effects. On the other hand, there is no significant relationship of facility outbreak size with the star rating, prior infection control violations, the Medicaid share, or the non-white share after accounting for the other controls and county fixed effects.

**Table 1:** Effect of staff tract measures and own tract measures on facility deaths per bed.

	(1)	(2)	(3)	(4)	(5)	(6)
Staff tract pop density		1.307*** (0.331)				
NH tract pop density		-0.139 (0.141)				
Staff tract pubtrans use			1.362*** (0.347)			
NH tract pubtrans use			-0.0890 (0.177)			
Staff tract share nonwhite				0.857*** (0.235)		
NH tract share nonwhite				-0.0589 (0.127)		
Staff tract pov rate					0.519** (0.168)	
NH tract pov share					-0.153 (0.0945)	
Staff tract share frontline						0.194 (0.201)
NH tract share frontline						-0.177 (0.111)
For-profit	0.525** (0.193)	0.504** (0.193)	0.522** (0.193)	0.522** (0.194)	0.510** (0.193)	0.516** (0.194)
Chain	0.348* (0.163)	0.380* (0.163)	0.370* (0.163)	0.360* (0.163)	0.365* (0.163)	0.347* (0.163)
Overall Rating	0.0468 (0.0916)	0.0474 (0.0915)	0.0478 (0.0915)	0.0529 (0.0917)	0.0530 (0.0916)	0.0471 (0.0916)
No prior infection viol.	0.257 (0.196)	0.260 (0.196)	0.256 (0.196)	0.249 (0.196)	0.251 (0.196)	0.260 (0.196)
Medicaid share	-0.0147 (0.0909)	0.00419 (0.0909)	0.000398 (0.0908)	-0.00875 (0.0908)	-0.0257 (0.0910)	-0.00771 (0.0915)
Resident share nonwhite	-0.0680 (0.118)	-0.171 (0.121)	-0.218 (0.124)	-0.282* (0.138)	-0.161 (0.125)	-0.0574 (0.120)
Avg severity	-0.0811 (0.0852)	-0.0693 (0.0852)	-0.0548 (0.0853)	-0.0463 (0.0856)	-0.0517 (0.0858)	-0.0761 (0.0855)
Occupancy Rate	0.681*** (0.0886)	0.668*** (0.0885)	0.673*** (0.0884)	0.681*** (0.0885)	0.676*** (0.0886)	0.681*** (0.0886)
25–50 beds	0 (.)	0 (.)	0 (.)	0 (.)	0 (.)	0 (.)
50–100 beds	0.486 (0.329)	0.506 (0.328)	0.538 (0.328)	0.515 (0.328)	0.498 (0.329)	0.485 (0.329)
100–150 beds	1.047** (0.334)	1.066** (0.333)	1.107*** (0.333)	1.093** (0.334)	1.062** (0.333)	1.048** (0.334)
150–200 beds	1.688*** (0.369)	1.670*** (0.369)	1.712*** (0.369)	1.679*** (0.369)	1.675*** (0.369)	1.676*** (0.369)
200+ beds	1.156** (0.410)	1.114** (0.410)	1.164** (0.409)	1.137** (0.410)	1.151** (0.410)	1.152** (0.410)
Constant	2.164*** (0.344)	2.181*** (0.344)	2.132*** (0.344)	2.166*** (0.344)	2.164*** (0.344)	2.172*** (0.344)
Fixed Effects	County	County	County	County	County	County
Depvar mean	3.735	3.735	3.735	3.735	3.735	3.735
Adj R2	0.29	0.29	0.29	0.29	0.29	0.29
N	6132	6132	6132	6132	6132	6132

This table reports OLS regression estimates of facility deaths per 100 beds on a collection of facility characteristics and county fixed effect. All continuous variables are normalized to have a standard deviation of 1. Standard errors are reported in parentheses. Significance

* *p* < .05

** *p* < .01

*** *p* < .001.

Columns (2)-(6) add characteristics of the staff and nursing home neighborhoods. The population density (column 2), public transportation use (column 3), and non-white share (column 4) of staff neighborhoods are all highly and statistically significantly associated with facility deaths per bed. For example, a one standard deviation increase in the average staff neighborhood population density is associated with an additional 1.3 deaths per 100 beds (p < .001, column 2); and comparably sized changes in staff neighborhood public transportation use, nonwhite share, and poverty rate are associated with an additional 1.4 (p < .001), 0.9 (p < .001), and 0.5 (p < .001) deaths per 100 beds, respectively (columns 3–5). In all of these cases, the same characteristic measured for the nursing home’s census tract is not statistically different from zero when the staff neighborhood variable is included. Column 6 shows no effect of the share of workers who are predicted to be frontline workers. The magnitudes of the effects in columns (2)-(4) are larger than any of the effects of other facility characteristics except facility size, suggesting that local staff geography may be an incredibly important factor in determining facility outbreaks. Most of the relationships from column (1) are unchanged by the inclusion of these neighborhood variables: size, for-profit status, and chain status continue to have significant effects on facility outbreaks. The one exception is the resident non-white share, which becomes more negatively correlated with facility deaths once staff neighborhood characteristics are added in columns (2)-(5).

A likely interpretation of the results in Table 1 is that nursing home staff members are an important source of infection, and that the identified neighborhood characteristics are good proxies for the level of community spread in a neighborhood. Unfortunately, few states report infection rates at the neighborhood level (counties are the lowest level of geography that is available nationally). Table 2 uses a subsample of eight states that provide more local data on case rates to test this interpretation. Columns (1) and (3) reproduce columns (3) and (4) (public transportation use and non-white share) from Table 1 for this smaller subsample, since these variables exhibited the strongest associations with facility infection in the full sample (because population density is highly correlated with public transportation use (ρ = .92), it is omitted for the sake of brevity). Both effects are still positive and highly significant predictors of facility death rates in this smaller subsample. Columns (2) and (4) add measures of the population case rate of COVID-19 in staff towns and the nursing home’s town (again normalized to have standard deviations of 1). A one standard deviation in the average infection rate of staff towns is associated with an additional 2.2 (p < .001, column 2) or 2.4 deaths per 100 beds (p < .001, column 4) at a facility. The infection rate of the nursing home’s town is associated with a smaller, but still large, increase in deaths. After including these measures, the estimated effects on staff neighborhood public transportation use and staff neighborhood non-white share are significantly reduced, suggesting that it is quite possible that those effects operate through differences in community-level infection.

**Table 2:** Relationship of nursing home deaths with local infection rates in staff and nursing home neighborhoods.

	(1)	(2)	(3)	(4)
Staff tract pubtrans use	1.412*** (0.303)	0.924** (0.316)		
Staff tract share nonwhite			1.345*** (0.354)	0.604 (0.380)
Staff town avg case rate		2.190*** (0.452)		2.330*** (0.466)
NH town case rate		0.546*** (0.159)		0.532*** (0.160)
For-profit	0.145 (0.361)	0.277 (0.357)	0.208 (0.362)	0.315 (0.358)
Chain	0.510 (0.302)	0.480 (0.298)	0.422 (0.302)	0.420 (0.298)
Overall Rating	-0.0483 (0.173)	-0.0525 (0.171)	-0.0165 (0.174)	-0.0372 (0.171)
No prior infection viol.	0.367 (0.355)	0.404 (0.352)	0.369 (0.356)	0.416 (0.352)
Medicaid share	0.0245 (0.183)	0.0126 (0.181)	0.00831 (0.183)	-0.000745 (0.181)
Resident share nonwhite	-0.230 (0.220)	-0.481* (0.222)	-0.383 (0.243)	-0.507* (0.241)
Avg severity	0.369* (0.150)	0.402** (0.149)	0.387* (0.152)	0.398** (0.150)
Occupancy Rate	0.814*** (0.174)	0.792*** (0.172)	0.829*** (0.174)	0.804*** (0.172)
25–50 beds	0 (.)	0 (.)	0 (.)	0 (.)
50–100 beds	1.204 (0.669)	1.171 (0.661)	1.107 (0.670)	1.104 (0.662)
100–150 beds	2.220*** (0.658)	2.199*** (0.650)	2.162** (0.659)	2.145** (0.651)
150–200 beds	2.682*** (0.712)	2.599*** (0.704)	2.516*** (0.713)	2.491*** (0.704)
200+ beds	2.077** (0.805)	1.997* (0.795)	1.948* (0.807)	1.924* (0.797)
Constant	1.995** (0.684)	1.846** (0.676)	2.116** (0.684)	1.923** (0.677)
Fixed Effects	County	County	County	County
Depvar mean	4.488	4.488	4.488	4.488
Adj R2	0.34	0.35	0.33	0.35
N	2038	2038	2038	2038

This table adds measures of town-level infection rates to the models in Table 1. The sample is all nursing homes in the subsample of eight states where this data is reported (CT, FL, IL, LA, MA, MD, RI, SC). All continuous variables have been normalized to have a standard deviation of 1. Standard errors in parentheses

* p < .05

** p < .01

*** p < .001.

Another interpretation is that these results are driven by the individual risk factors of staff members themselves, rather than their neighborhoods. This could have different policy implications, such as suggesting that nursing homes could potentially be protected if we provided staff with non-public transportation options. However, data from the American Community Survey suggests that very few (less than 5%) nursing home workers take public transportation to work in the study states (S2 Table). Furthermore, after controlling for the overall neighborhood measure, there is no effect of a measure of neighborhood public transportation use that is restricted to workers in the education and health care industry (S3 Table, column 1). Likewise, after controlling for the neighborhood racial composition, there is no effect of the share of workers who work on the same block as the nursing home who are non-white, which should be a better proxy for the racial composition of a nursing home’s staff (S3 Table, column 2).

It is possible that the initial infection of a facility and the containment or spread of the virus at the facility are affected by different factors. To study this, S4 Table reproduces the main results using a binary indicator for an outbreak, whether or not a facility reported any death, rather than the continuous measure used throughout the paper. Columns (2) and (3) show that the main results apply when investigating the presence of an outbreak: staff neighborhood characteristics continue to be one of the most important predictors of facility infection, and there continues to be a large effect of for-profit status. There are some differences: the effect of chain status is not significant here, and there is a slightly negative effect of star rating on the binary measure, and a positive effect of the resident non-white share, suggesting that lower-rated facilities and facilities with more non-white residents were more likely to experience an outbreak, even though outbreak size was not correlated with these characteristics.

These results beg the question: what types of facilities are likely to have staff who live in more dense and nonwhite neighborhoods with more public transportation? Are lower-quality, for-profit, nursing homes more likely to have higher staff neighborhood exposure? Are the most exposed facilities also the ones with the lowest wages, or the most non-white residents? If so, these results could offer a mechanism to explain systematically higher deaths at facilities without unions or facilities with fewer white residents that has been documented in other literature [7,10]. Fig 3 shows partial correlations of the staff neighborhood measures with other facility characteristics, controlling for county fixed effects, with a particular focus on measures related to a facility’s staffing practices. Both staff neighborhood public transportation use and staff neighborhood non-white share are positively correlated with larger, for-profit, and lower-rated facilities, but the correlations are relatively small. Likewise, there is a small negative correlation of the wage paid to certified nursing assistants (40% of the nursing home workforce, and the occupation likely to have the most contact with patients). On the other hand, staff exposure exhibits much larger correlations with the demographics of the residents: facilities with more Medicaid patients, and especially more nonwhite patients, are more likely to have higher measures of the staff neighborhood exposure measures. These results explain why in Table 1, the resident nonwhite share coefficient becomes insignificant after including the staff neighborhood measures, and suggest a potential channel for observed racial disparities in COVID infection across nursing homes: nursing homes with more non-white residents appear to employ more staff from the most highly exposed neighborhoods.

## Discussion

This study uses a novel approach to measuring staff neighborhood characteristics to provide new evidence that the local geography of where staff lives is a strong predictor of nursing home outbreak sizes, even after controlling for a nursing home’s county. Specifically, nursing homes whose staff come from denser, less white neighborhoods with more public transportation use have had significantly larger outbreaks of COVID-19, and that these measures are much more powerful in explaining differences in death rates within a county than many other facility characteristics (such as nursing home rating), and also than the same characteristics of the nursing home’s own neighborhood.

While there were early efforts to close nursing homes to visitors and protect nursing home residents, the experience of these homes has indicated that these efforts were not nearly enough, with significant numbers of homes becoming infected after they were “locked down.” Because of the close personal contact they have with residents, staff members are a likely source of transmission, and these results lend support to this hypothesis that the spread of COVID-19 in staff communities was an important mechanism driving facility outbreaks in the first wave of the pandemic. However, the small effect of facility management variables compared to the large effect of staff neighborhood characteristics suggests that it may ultimately be necessary to control outbreaks in the community in order to control facility outbreaks. It is possible that these relationships are specific to the first wave of deaths in the spring and early summer of 2020, and that as nursing homes gained experience with controlling outbreaks, other variables became more important in determining deaths from COVID-19. While this study does not investigate deaths in the later months of the pandemic, descriptive evidence from other researchers appears to indicate that community spread remained important after the first wave [14].

Previous research has documented substantial segregation in long-term care, and how the location of high-quality facilities may exacerbate other inequalities [15–19]. In the case of COVID-19, even though this study does not find evidence of a significant effect of facility rating on facility outbreaks, it does uncover the concerning finding that the facilities that employ staff from neighborhoods that are more exposed to COVID-19 infection are also the facilities that serve more non-white residents. Early evidence suggests that black and Latino communities have been hit hardest by the pandemic [20–24]. The fact that the nursing home industry draws staff disproportionately from these communities in general may explain some of the enormous impact of the pandemic on nursing homes.

Finally, the persistent and relatively large effect of for-profit status on COVID-19 outbreaks as well as the consistently small or zero effect of a facility’s star rating (after accounting for geography and other facility characteristics) both merit further study, as it suggests that non-profit homes may have responded differently to the pandemic (for instance, in the use or availability of PPE or surveillance testing), but that the rating system was not able to predict these differences. In addition, the fact that wages are not highly correlated with high staff exposure also offers an opportunity for future study to understand why certain homes employ more heavily from more exposed communities; it may indicate that these nursing homes are most conveniently located for people living in these communities, or that facilities with more and less non-white residents have different hiring practices.

This study has a few limitations. First, it is important to note that the analysis in this study is correlational and there may therefore be omitted variables that are driving the results. For example, S5 Table shows that the coefficients on staff neighborhood characteristics are reduced if the distance of the nursing home to the central business district of the nearest metropolitan area is included, though they remain relatively large and statistically significant. This could either mean that (1) staff neighborhood characteristics are a true risk factor for facility outbreaks, and nursing homes that are more centrally located are simply likelier to draw staff from more exposed neighborhoods, or (2) centrality affects facility outbreaks through other mechanisms besides staff neighborhoods. However, in this case, the fact that staff neighborhood characteristics continue to be significant after controlling for centrality suggest that the former may be more important, and also offer a lower bound for these effects. A second limitation is that I use the neighborhood characteristics of all service sector employees on the same block as the nursing home to proxy for the neighborhood characteristics of nursing home staff, and that the block-level data I use to identify staff neighborhoods is from 2017 (the most recent data available at the time of analysis). S6 Table shows that the results are not significantly affected by excluding nursing homes whose blocks contain significantly more service sector employees than my prediction of the nursing home’s employment. In addition, measurement error in staff neighborhood characteristics is likely to attenuate the observed relationship.

Finally, because the analysis is within counties, it cannot offer much insight into the effects of different county- or state-level policies in the COVID-19 response, though these are likely to have been important in determining infection rates and deaths. Additionally, although Table 2 offers suggestive evidence that the relationship between staff neighborhood characteristics and can be explained by higher infection rates in these neighborhoods, it is limited by the lack of data on infection rates by neighborhood (only one state in the sample provides case-level data at the tract-level, the remaining states in Table 2 provided data at the town or zip level, and the other states not included in Table 2 only report case data at the county level). More granular data of this form would help confirm the hypotheses of this study.

### Conclusions

During the first wave of the pandemic, which nursing homes experienced the largest outbreaks of COVID-19 within a county was not random, but it was also not largely determined by other measures of quality commonly cited in the nursing home literature, such as star rating. Instead, a key determinant was the characteristics of the neighborhoods where nursing home staff members lived—facilities whose staff lived in denser, less white neighborhoods with more public transportation use have had significantly more deaths than other facilities in the same county.

## Supporting information

S1 Fig (TIF)

S1 Table Summary statistics for analysis sample. (DOCX)

S2 Table Characteristics of nursing home workers from American Community Survey and BLS data. (DOCX)

S3 Table Relationship of facility deaths per bed with proxies for staff characteristics and staff neighborhood characteristics. (DOCX)

S4 Table Relationship of binary measure of facility infection (any death) with facility and neighborhood characteristics. (DOCX)

S5 Table Relationship of facility deaths per bed with distance to central business district and staff and nursing home neighborhood characteristics. (DOCX)

S6 Table Subsample analysis of relationship of facility deaths per bed with staff neighborhood characteristics in sample excluding nursing homes whose blocks have significant additional service sector employment. (DOCX)

S1 File Data appendix. (PDF)
