# Supplementary material for: Relationship between nursing home COVID-19 outbreaks and staff neighborhood characteristics
Source: PLoS One. 2022 Apr 19;17(4):e0267377. doi: 10.1371/journal.pone.0267377 (PMC9017897; doi:10.1371/journal.pone.0267377)
Supplement: S1 Table — (DOCX) [file pone.0267377.s002.docx]

| **Variable** | **Mean** | **Std. Dev.** |
| --- | --- | --- |
| Number of beds | 119 | 67 |
| For-profit | .73 |  |
| Non-profit | .24 |  |
| Public | .03 |  |
| Chain | .58 |  |
| Star rating | 3.1 | 1.4 |
| - Inspection rating | 2.7 | 1.2 |
| - Staffing rating | 2.9 | 1.1 |
| - Quality measure rating | 3.8 | 1.2 |
| RN wage | 34.3 | 6.4 |
| CNA wage | 15.0 | 2.7 |
| Occupancy rate | .84 | .13 |
| Medicaid share | .60 | .23 |
| Resident share non-white | .23 | .23 |
| Staff tract pop density (pp/sq mi) | 4906 | 7386 |
| Staff tract pub trans use | .05 | .08 |
| Staff tract share nonwhite | .28 | .17 |
| Staff tract pov rate | .19 | .07 |
| Staff tract share frontline | .31 | .03 |
| NH tract pop density (pp/sq mi) | 4543 | 9484 |
| NH tract pub trans use | .05 | .10 |
| NH tract share nonwhite | .25 | .22 |
| NH tract pov rate | .18 | .17 |
| NH tract share frontline | .30 | .06 |
