# Supplementary material for: Relationship between nursing home COVID-19 outbreaks and staff neighborhood characteristics
Source: PLoS One. 2022 Apr 19;17(4):e0267377. doi: 10.1371/journal.pone.0267377 (PMC9017897; doi:10.1371/journal.pone.0267377)
Supplement: S2 Table — (DOCX) [file pone.0267377.s003.docx]

| Top Occupations (OES) | Share |
| --- | --- |
| - Certified Nursing Assistant | .38 |
| - Licensed Practical Nurses | .13 |
| - Registered Nurses | .10 |
| - Food preparation and serving | .10 |
| - Building cleaners | .10 |
| - Office and administrative support | .05 |
| - Other healthcare practitioners (therapists, etc.) | .03 |
| - Laundry workers | .02 |
| - Other | .09 |
|  |  |
| Demographics (ACS) |  |
| - Female | .84 |
| - White non-hispanic | .55 |
| - Black | .27 |
| - Hispanic or Latino | .06 |
| - High school or less | .38 |
| - Some college | .27 |
| - Two year degree | .15 |
| - Four year degree or more | .19 |
| - Commute to work by car | .92 |
| - Commute to work on public transportation | .04 |
| - Annual wage/salary income < $30,000 | .59 |
| - Annual wage/salary income < $50,000 | .82 |
