# Supplementary material for: Relationship between nursing home COVID-19 outbreaks and staff neighborhood characteristics
Source: PLoS One. 2022 Apr 19;17(4):e0267377. doi: 10.1371/journal.pone.0267377 (PMC9017897; doi:10.1371/journal.pone.0267377)
Supplement: S3 Table — (DOCX) [file pone.0267377.s004.docx]

|  | (1) | (2) |
| --- | --- | --- |
| Staff tract pubtrans use | 1.159^***^ (0.266) |  |
| Staff tract PT share (Ed/Health) | 0.0465 (0.139) |  |
| Staff tract share nonwhite |  | 1.078^***^ (0.317) |
| Staff on block share nonwhite |  | -0.238 (0.208) |
| For-profit | 0.518^**^ (0.193) | 0.541^**^ (0.193) |
| Chain | 0.375^*^ (0.163) | 0.363^*^ (0.163) |
| Star rating | 0.0476 (0.0915) | 0.0470 (0.0916) |
| No prior infection viol. | 0.254 (0.195) | 0.253 (0.196) |
| Medicaid share | -0.00697 (0.0880) | -0.00784 (0.0880) |
| Resident share nonwhite | -0.214 (0.121) | -0.295^*^ (0.130) |
| Avg severity | -0.0597 (0.0813) | -0.0516 (0.0817) |
| Occupancy Rate | 0.664^***^ (0.0871) | 0.672^***^ (0.0871) |
| 25-50 beds | 0 (.) | 0 (.) |
| 50-100 beds | 0.540 (0.328) | 0.531 (0.328) |
| 100-150 beds | 1.109^***^ (0.333) | 1.115^***^ (0.333) |
| 150-200 beds | 1.732^***^ (0.368) | 1.717^***^ (0.368) |
| 200+ beds | 1.163^**^ (0.409) | 1.155^**^ (0.409) |
| Constant | 2.053^***^ (0.343) | 2.037^***^ (0.344) |
| fe | County | County |
| ymean | 3.735 | 3.735 |
| r2_a | 0.29 | 0.29 |
| N | 6146 | 6146 |

Standard errors in parentheses

^*^ *p* < .05, ^**^ *p* < .01, ^***^ *p* < .001
