# Supplementary material for: Relationship between nursing home COVID-19 outbreaks and staff neighborhood characteristics
Source: PLoS One. 2022 Apr 19;17(4):e0267377. doi: 10.1371/journal.pone.0267377 (PMC9017897; doi:10.1371/journal.pone.0267377)
Supplement: S4 Table — (DOCX) [file pone.0267377.s005.docx]

|  | (1) | (2) | (3) |
| --- | --- | --- | --- |
|  |  |  |  |
| Staff tract pubtrans use |  | 0.0888^***^ (0.0249) |  |
| NH tract pubtrans use |  | -0.0245 (0.0127) |  |
| Staff tract share nonwhite |  |  | 0.0638^***^ (0.0169) |
| NH tract share nonwhite |  |  | -0.0121 (0.00913) |
| For-profit | 0.0404^**^ (0.0139) | 0.0396^**^ (0.0139) | 0.0393^**^ (0.0139) |
| Chain | 0.0183 (0.0117) | 0.0190 (0.0117) | 0.0189 (0.0117) |
| Overall Rating | -0.0131^*^ (0.00658) | -0.0129^*^ (0.00658) | -0.0124 (0.00659) |
| No prior infection viol. | 0.0119 (0.0141) | 0.0116 (0.0141) | 0.0114 (0.0141) |
| Medicaid share | -0.00554 (0.00653) | -0.00496 (0.00653) | -0.00517 (0.00652) |
| Resident share nonwhite | 0.0292^***^ (0.00845) | 0.0237^**^ (0.00890) | 0.0173 (0.00992) |
| Avg severity | -0.0229^***^ (0.00612) | -0.0217^***^ (0.00613) | -0.0206^***^ (0.00615) |
| Occupancy Rate | 0.0208^**^ (0.00636) | 0.0203^**^ (0.00636) | 0.0208^**^ (0.00635) |
| 25-50 beds | 0 (.) | 0 (.) | 0 (.) |
| 50-100 beds | 0.127^***^ (0.0236) | 0.130^***^ (0.0236) | 0.129^***^ (0.0236) |
| 100-150 beds | 0.231^***^ (0.0240) | 0.233^***^ (0.0240) | 0.234^***^ (0.0240) |
| 150-200 beds | 0.301^***^ (0.0265) | 0.301^***^ (0.0265) | 0.300^***^ (0.0265) |
| 200+ beds | 0.326^***^ (0.0295) | 0.325^***^ (0.0294) | 0.324^***^ (0.0294) |
| Constant | 0.208^***^ (0.0247) | 0.207^***^ (0.0247) | 0.209^***^ (0.0247) |
| Fixed Effects | County | County | County |
| Depvar mean | 0.455 | 0.455 | 0.455 |
| Adj R2 | 0.40 | 0.40 | 0.40 |
| N | 6132 | 6132 | 6132 |

Standard errors in parentheses

^*^ *p* < .05, ^**^ *p* < .01, ^***^ *p* < .001
