# Supplementary material for: Relationship between nursing home COVID-19 outbreaks and staff neighborhood characteristics
Source: PLoS One. 2022 Apr 19;17(4):e0267377. doi: 10.1371/journal.pone.0267377 (PMC9017897; doi:10.1371/journal.pone.0267377)
Supplement: S5 Table — (DOCX) [file pone.0267377.s006.docx]

|  | (1) | (2) |
| --- | --- | --- |
| Distance to CBD | -0.978^***^ (0.251) | -1.052^***^ (0.244) |
| Staff tract pubtrans use | 0.799^*^ (0.336) |  |
| NH tract pubtrans use | -0.0297 (0.163) |  |
| Staff tract share nonwhite |  | 0.569^*^ (0.244) |
| NH tract share nonwhite |  | -0.0255 (0.126) |
| For-profit | 0.529^**^ (0.193) | 0.530^**^ (0.193) |
| Chain | 0.374^*^ (0.162) | 0.370^*^ (0.162) |
| Star rating | 0.0500 (0.0915) | 0.0504 (0.0916) |
| No prior infection viol. | 0.263 (0.195) | 0.264 (0.195) |
| Medicaid share | 0.0357 (0.0885) | 0.0291 (0.0884) |
| Resident share nonwhite | -0.290^*^ (0.123) | -0.345^*^ (0.137) |
| Avg severity | -0.0673 (0.0813) | -0.0613 (0.0816) |
| Occupancy Rate | 0.656^***^ (0.0876) | 0.660^***^ (0.0876) |
| 25-50 beds | 0 (.) | 0 (.) |
| 50-100 beds | 0.537 (0.328) | 0.528 (0.328) |
| 100-150 beds | 1.116^***^ (0.333) | 1.114^***^ (0.333) |
| 150-200 beds | 1.680^***^ (0.369) | 1.663^***^ (0.368) |
| 200+ beds | 1.117^**^ (0.409) | 1.096^**^ (0.409) |
| Constant | 2.092^***^ (0.343) | 2.095^***^ (0.343) |
| fe | County | County |
| ymean | 3.736 | 3.737 |
| r2_a | 0.29 | 0.29 |
| N | 6141 | 6142 |
