# Supplementary material for: Relationship between nursing home COVID-19 outbreaks and staff neighborhood characteristics
Source: PLoS One. 2022 Apr 19;17(4):e0267377. doi: 10.1371/journal.pone.0267377 (PMC9017897; doi:10.1371/journal.pone.0267377)
Supplement: S6 Table — (DOCX) [file pone.0267377.s007.docx]

|  | (1) |  | (2) |  |
| --- | --- | --- | --- | --- |
| Staff tract pubtrans use | 1.032^*^ | [0.424] |  |  |
| NH tract pubtrans use | 0.055 | [0.214] |  |  |
| Staff tract share nonwhite |  |  | 0.975^**^ | [0.304] |
| NH tract share nonwhite |  |  | -0.152 | [0.163] |
| For-profit | 0.327 | [0.274] | 0.328 | [0.274] |
| Chain | 0.349 | [0.215] | 0.332 | [0.214] |
| Overall Rating | 0.140 | [0.114] | 0.151 | [0.114] |
| No prior infection viol. | 0.328 | [0.251] | 0.325 | [0.251] |
| Medicaid share | -0.124 | [0.131] | -0.137 | [0.131] |
| Resident share nonwhite | -0.194 | [0.157] | -0.265 | [0.180] |
| Occupancy Rate | 0.661^***^ | [0.128] | 0.674^***^ | [0.127] |
| 25-50 beds | 0.000 | [.] | 0.000 | [.] |
| 50-100 beds | 0.873 | [0.554] | 0.869 | [0.554] |
| 100-150 beds | 1.481^**^ | [0.552] | 1.482^**^ | [0.552] |
| 150-200 beds | 2.157^***^ | [0.582] | 2.118^***^ | [0.582] |
| 200+ beds | 1.646^**^ | [0.622] | 1.616^**^ | [0.622] |
| Constant | 2.030^***^ | [0.584] | 2.085^***^ | [0.584] |
| Fixed Effects | County |  | County |  |
| Depvar mean | 3.967 |  | 3.967 |  |
| Adj R2 | 0.27 |  | 0.27 |  |
| N | 3991 |  | 3991 |  |
